# 

**Published:** 1882-11-20

**Authors:** 


HILDEBRANDT, the great gynecological teacher of Konningeburg, is dead.

ETHER VS. CHLOROFORM.—The number of deaths thus far recorded of chloroform is 368, while those from ether are only 27.

THE American Gynecological Association will hold its next meeting in Philadelphia on the third Tuesday in September, 1883.

MEDICAL JOURNALS.—It is said that the number of Medical Journals published in the City of Philadelphia falls but little short of the entire number published in the whole of England.

THE Order of Disorder in Mental Disease, by O. Everts, M. D., superintendent of the Cincinnati Sanitarium. This is a well written, able and instructive paper on the subject treated.

### *RECEIPTED.*

1881.—Drs. B M Walker, E P Overby, —— McCallum. A A Stanley.

1882.—Drs. W Cusick, P M Catching, G M McMillan, A J Pinson, B E Clark, M B Pollard, W S Glass, H J Walker, W H Peek, J B Rutland, Richard Inge, M V Harrington, T N Skeen, Jno. G Moore, J W Unger.

1883.—Drs. L M Wood, to Oct., H Nance, W H Boyken, A B Coleman, T S Jones.

